# The Role of Cannabinoids in Advancing Cancer Treatment: Insights from Evidence-Based Medicine

**DOI:** 10.1007/s11912-024-01589-4

**Published:** 2024-08-07

**Authors:** Magdalena Skórzewska, Katarzyna Gęca

**Affiliations:** Department of Surgical Oncology, Medical University of Lublin, Radziwiłłowska 13 St, 20-080 Lublin, Poland

**Keywords:** Cannabinoids, THC, CBD, Evidence-Based Medicine, EBM, Cancer Treatment

## Abstract

**Purpose of Review:**

This document critically examines the role of cannabinoids in cancer care during an era marked by rapid advancements in oncology and changing perceptions on cannabis. It traces the historical context of cannabis in medicinal use, navigating its journey from widespread acceptance, subsequent criminalization, to its resurgence in modern therapeutic applications, particularly within the framework of Evidence-Based Medicine (EBM).

**Recent Findings:**

Anchored in EBM principles, this study synthesizes current research from clinical trials, systematic reviews, and meta-analyses to evaluate the efficacy and safety of cannabinoids in oncology. The focus is on their palliative effects, considering the nuances of effectiveness, risk assessment, and challenges inherent in translating these findings into clinical guidelines.

**Summary:**

The study seeks to bridge the gap between scientific research and clinical practice, offering insights to inform future oncological therapies and symptom management strategies involving cannabinoids. The potential benefits and risks of cannabinoid use in cancer treatment are assessed to guide clinicians and researchers in developing comprehensive, evidence-based approaches to patient care.

## Introduction

Cannabinoid medications showcase the therapeutic potential of cannabinoids in treating a range of conditions. However, their use is subject to regulatory approval and medical guidance, as they can have significant side effects and interactions. The development and use of cannabinoid medications continue to be an area of active research, with ongoing studies aimed at exploring new therapeutic applications and understanding their mechanisms of action more fully.

The historical use of cannabis for medicinal purposes dates back to at least 2700 years B.C., when Chinese doctors employed it to treat malaria, rheumatic pain, and various other diseases, in addition to its use as an anaesthetic [1]. In 1839, W.B. O'Shaughnessy, a surgeon working for the British East India Company, introduced it into Western medicine after recognising its medicinal properties while in India. Its application was advocated for its reported analgesic, sedative, anti-inflammatory, antispasmodic, and anticonvulsant effects [2]. "Herbal cannabis" typically refers to the dried buds of the female Cannabis Sativa L plant, which have the highest levels of natural cannabinoids. These include primarily Δ9-THC and cannabidiol (CBD), along with others such as cannabigerol, cannabichromene, cannabidivarin, and tetrahydrocannabivarin [3].

The global trend towards the acceptance of medicinal cannabis has resulted in the legalization of cannabis for medical purposes in 70 countries. Out of these, 26 countries have legalized the use of high- Tetrahydrocannabinol (THC) cannabis for medical purposes. In 2020, consumers spent an estimated $415 billion on high-THC cannabis, highlighting the substantial global market. According to projections, this figure is expected to reach $496 billion by 2025. Nonetheless, a substantial amount of this demand (94% in 2020) continues to exist in illicit or unregulated markets. In comparison, the sales of high-THC cannabis through legal regulated markets amounted to $23.7 billion in 2020 and are forecasted to rise to $51 billion by 2025 [4].

Regarding applications, the chronic pain segment accounted for a substantial portion of the revenue share in 2022, constituting 25.9%. This suggests a rising recognition of medical cannabis as a treatment option for chronic and neuropathic pain. There is an expected substantial expansion in the market for medical marijuana. The driving force behind this growth is the heightened awareness of therapeutic applications, changing legal landscape, and the escalating number of clinical trials assessing the effectiveness of cannabis in pain management [5].

The utilization of cannabinoids, obtained from the Cannabis sativa plant, has shown promise in palliative care as a potential option for managing symptoms in patients with life-limiting illnesses. The therapeutic potential of cannabinoids is primarily ascribed to their interaction with the endocannabinoid system (ECS) in the body, which plays a crucial role in regulating pain, mood, and other physiological responses [6]. Effective pain management holds significant importance in the realm of palliative care. It has been demonstrated, that inhaled cannabis consistently proves effective in reducing chronic non-cancer pain, while oral cannabinoids have the potential to improve certain aspects of chronic pain, such as sleep and overall quality of life (QoL). Nonetheless, the effectiveness of these treatments varies depending on the type of pain and how they are administered [7]. The "entourage effect," which refers to the enhanced combined effect of cannabinoids and terpenes, aromatic compounds present in the Cannabis sativa plant, has been brought to light by recent research [8]. The synergy between these factors may amplify the pain-relieving attributes of cannabinoids, potentially providing a more effective method for pain management in palliative care [9].

Although there are potential benefits, the utilization of cannabinoids in palliative care presents certain difficulties. Research has demonstrated varying degrees of efficacy and a spectrum of adverse effects (AEs). Additionally, the evidence quality from trials frequently diminishes due to high or uncertain risks of bias. Hence, it is imperative to conduct further high-quality, randomized controlled trials (RCT) in order to establish more precise guidelines for the application of cannabinoids in palliative care settings [10].

The aim of this review is to provide a comprehensive and forward-looking perspective on the potential role of cannabinoids and its palliative effects exclusively in the oncological patients.

### Endocannabinoid System in *Cancer*—Key Roles in Immune Modulation and Tumor Dynamics

The ECS is a complex and intricate biological network that plays a critical role in maintaining physiological homeostasis. This system comprises endogenous lipid-based signaling molecules, known as endocannabinoids, predominantly anandamide (AEA) and 2-arachidonoylglycerol (2-AG), which are synthesized on demand from cell membrane phospholipids [11]. These endocannabinoids function as retrograde neurotransmitters, modulating synaptic activity [12]. The primary receptors of the ECS are the cannabinoid receptors type 1 (CB1) and type 2 (CB2). CB1 receptors, mainly found in the central nervous system, are instrumental in modulating neurotransmitter release and thereby influence pain perception, mood, and memory. Conversely, CB2 receptors are more prevalent in peripheral tissues, particularly within immune cells, and play a significant role in regulating immune responses and inflammation [13].

**Fig. 1:**
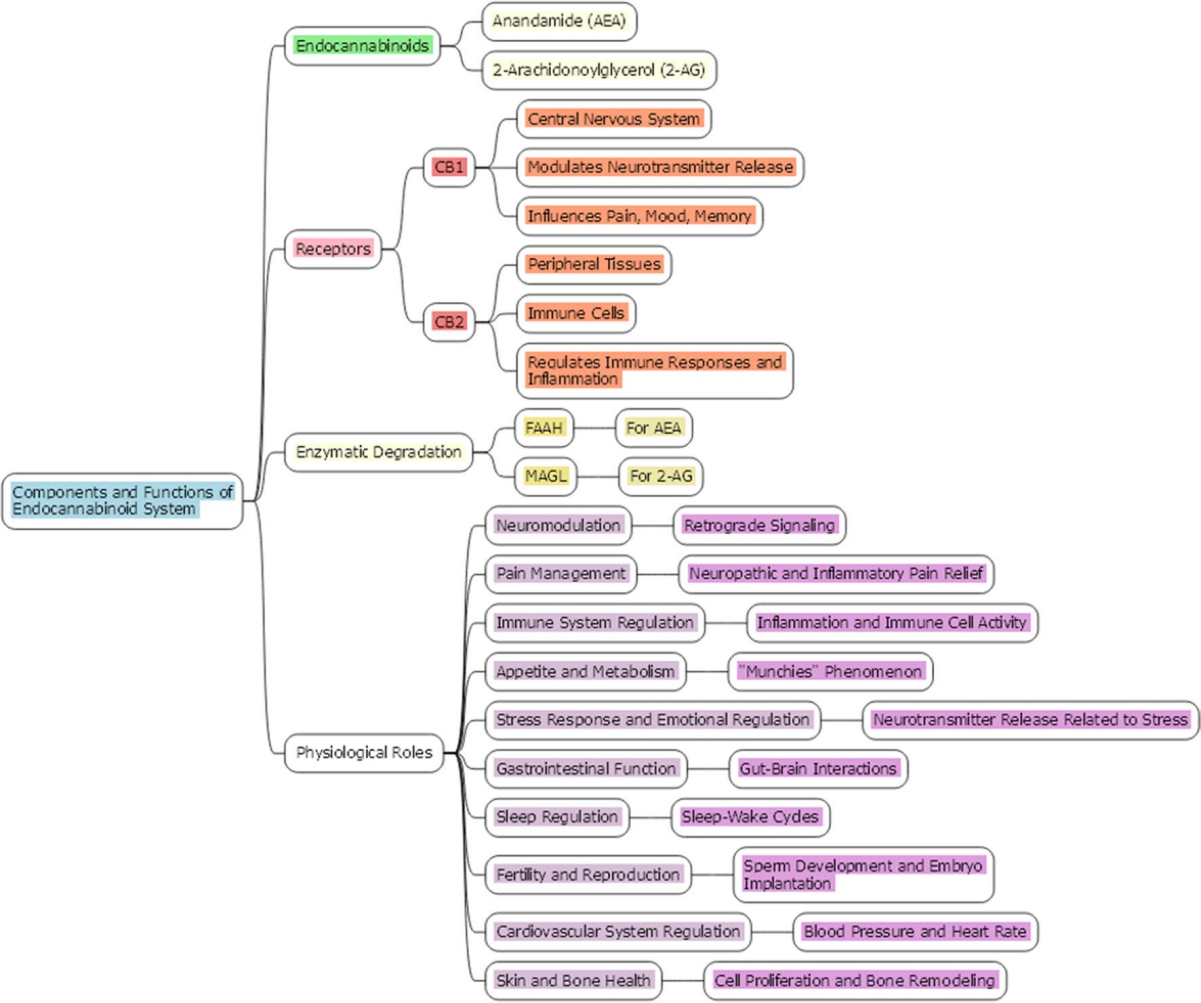
Components and functions of ECS

The enzymatic degradation of endocannabinoids is another crucial aspect of the ECS. Enzymes like fatty acid amide hydrolase (FAAH) for AEA and monoacylglycerol lipase (MAGL) for 2-AG ensure the transient nature of endocannabinoid signaling [13]. Recent advancements in ECS research have shed light on its diverse roles in human physiology. For instance, a study by Ratcliffe et al. explored the developmental impacts of cannabis smoke exposure on the ECS in the gastrointestinal tract. This research provides insight into the significant role of the ECS in the gut-brain axis, highlighting its influence on gastrointestinal functions [12]. Furthermore, investigations into the relationship between the ECS and metabolic flexibility, as well as its association with metabolic markers in middle-aged individuals, underscore the system's involvement in metabolic regulation [14]. Additionally, studies delving into the effects of cannabis on bowel motility contribute to our understanding of the ECS's comprehensive influence on gastrointestinal function [15]. The role of the ECS in modulating the composition of immune cells within the tumor microenvironment (TME) is crucial. This modulation has a significant impact on the functionality of immune cells and the development of tumor growth. These findings suggest that cannabinoids could have the potential for immunotherapy. However, to fully grasp these interactions, further investigation is necessary [16]. The main components and functions of ECS are presented in Fig. 1.

The ECS has been shown to alter the immune cell makeup in the TME across various tumor types, thereby influencing immune cell activity and, by extension, tumor growth [17–23]. Research primarily centered on in vitro studies has predominantly focused on how direct activation or inhibition of cannabinoid receptors (CB) expressed in cancer cells impacts their growth dynamics [24]. Nonetheless, the exact effects of cannabinoids on immune cells within tumors, and consequently on tumor development, remain largely unexplored.

The TME, apart from malignant cells, comprises a complex and varied assembly of both infiltrating and resident host cells, a range of secreted factors, and the extracellular matrix (ECM). While the TME composition varies across different tumor types, certain consistent features are observed in immune and stromal cells, blood vessels, and the ECM. It is a well-established fact that cells within the TME actively contribute to regulating cancer progression [25]. Both adaptive and innate immune cells, crucial components of the TME, play dual roles in either suppressing or promoting tumor growth. Within this environment, the adaptive immune response encompasses the activities of T cells, B cells, and Natural Killer (NK) cells, while the innate immune response is mediated through myeloid cells such as macrophages, neutrophils, eosinophils, dendritic cells (DCs), and other immune constituents [25].

The ECS components are universally found in all cellular components of tumor masses, including both host cells in the TME and the cancer cells themselves. This widespread presence poses significant challenges in pinpointing their precise role in tumor progression and leveraging them as therapeutic targets.

To date, the body of research examining the impact of endo-/cannabinoids and ECS components on the TME and their role in tumor development remains limited [26]. Study conducted by Kienzl et al. reported that CB2 receptors, derived from TME cells, foster tumor growth in a model of non-small cell lung cancer (NSCLC). This process involves the reduction in accumulation and cytotoxic activity of CD8 + T cells and NK cells. Furthermore, it was observed that a deficiency of CB2 receptors in host cells amplifies the expression of programmed death-1 (PD-1) and its ligand PD-L1 on lymphoid and myeloid cells [45].

The role of the ECS component, 2-AG, is also notable. In a pancreatic cancer model, Qiu et al. found that 2-AG suppresses tumor proliferation via activation of the CB1 receptor, rather than the CB2 receptor, and fosters the maturation of DC phenotypes and production of pro-inflammatory cytokines through up-regulation of the signal transducer and activator of transcription 6 (p-STAT6) [18]. Contrastingly, THC did not directly impact melanoma cell growth, but indirectly influenced tumor growth through interactions with TME cells in a CB receptor-dependent manner [19]. Specifically, THC inhibited the infiltration of pro-tumorigenic myeloid immune cells, showcasing the ECS's potential in modulating tumor growth through TME interactions.

Conversely, Xiang et al. demonstrated an opposing mechanism, where MGL in macrophages inhibited tumor progression mediated by CB2 receptors. They found that a deficiency in MGL enhanced macrophage activation in a CB2/TLR4-dependent manner, affecting the exhaustion status of CD8 + T cells in colon and breast cancer models [20].

Collectively, these studies indicate that ECS components like MGL and CB2 can influence tumor progression through their interactions with immune cells and immune checkpoints within the TME. However, it is crucial to note that the pro- or anti-tumorigenic effects of the ECS may vary depending on the cancer type.

## EBM on Cannabinoids in Oncology

The current status of cannabinoid research in oncology as of 2024 indicates an active and evolving field, with a focus on understanding the role of cannabinoids in cancer treatment and symptom management.

**Fig. 2:**
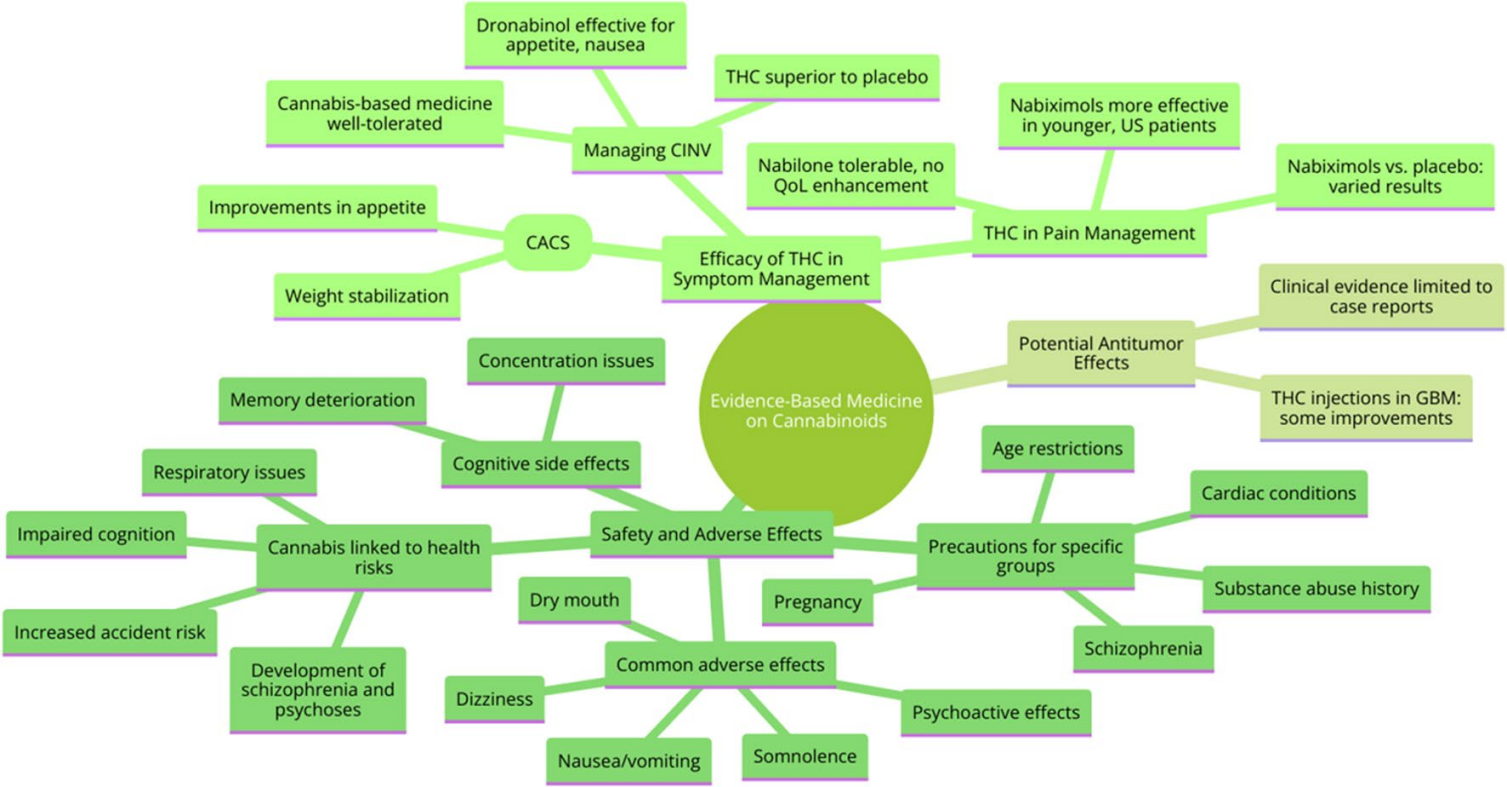
The applications of cannabinoids in oncological patients from EBM standpoint

The primary emphasis of Evidence-Based Medicine (EBM) in relation to the application of THC in oncology centers on the meticulous evaluation of clinical trials, systematic reviews, and meta-analyses. This approach aims to offer a definitive, evidence-based perspective on its effectiveness and safety [27, 28]. The applications of cannabinoids in oncological patients from EBM standpoint are presented in Fig. 2. Summarized information concerning approved cannabinoid-based medications in cancer management are presented in Table 1.

1. Chemotherapy-induced nausea and vomiting:

**Table 1:** Approved cannabinoid-based medications in cancer management

Indication	Drug (Trade name)			Guidelines	References
	Dronabinol (Marinol®, Syndros®)	Nabilone (Cesamet®, Cnemis®)	Nabiximols (Sativex®)		
Chemotherapy-induced nausea and vomiting (CINV)	FDA approved in 1985 for treatment of CINV, mostly after the failure of previous therapies	FDA approved in 1985 for treatment of CINV		NCCN and ASCO guidelines include the indication of Nabilone and Dronabinol for nausea and vomiting refractory to conventional therapy The recommended dosage for Dronabinol oral solution is 2.5–5 mg, to be taken every 4–6 h. As for Nabilone, a dosage of 1–2 mg can be taken twice daily as rescue therapy	[70–72]
Pain Management	No cannabinoid-based medications approved by FDA or EMA for the treatment of cancer pain		Approved for multiple sclerosis-associated neuropathic pain	ASCO, NCCN, and ESMO share similar perspectives on the clinical incorporation of cannabinoids for this purpose. In the context of cancer pain, clinicians should refrain from recommending cannabinoids, as they neither offer benefits nor pose any harm	[73]
Cancer-related Cachexia and Anorexia Syndrome (CACS)	Approved for anorexia associated with weight loss in patients with AIDS, but not for cancer patients			As per ASCO guidelines, the efficacy of cannabinoids for CACS is not supported due to the insufficient strength of evidence from existing research. Likewise, ESMO maintains that the current body of literature does not provide enough evidence to support the utilization of medical cannabis or its derivatives for the treatment of CACS. The NCCN guidelines incorporate the potential utilization of cannabinoids for appetite stimulation in patients with a significantly limited life expectancy (weeks, days until demise)	[51, 74]

FDA—U.S. Food and Drug Administration, EMA—European Medicines Agency, ASCO—American Society of Clinical Oncology, ESMO—European Society for Medical Oncology, NCCN—National Comprehensive Cancer Network, AIDS—acquired immunodeficiency syndrome.

Chemotherapy-induced nausea and vomiting (CINV) remain a significant challenge, despite advancements in anti-emetic treatments. THC, a principal component of cannabis, has shown potential in managing these symptoms. Sallan et al.'s foundational study highlighted THC's superiority over a placebo in alleviating CINV [29]. However, the efficacy of cannabis in treating CINV is not uniformly recognized. In some cases, patients have preferred enduring the side effects of CTH over those resulting from cannabis usage [30].

A German study indicated that Dronabinol, a synthetic form of THC, could be effective in treating loss of appetite and nausea associated with liver metastases from malignant melanoma. Although all patients in this study reported dizziness, the severity was not significant enough to warrant discontinuation of the medication. However, the inadequate sample size of this study restricts the broader applicability of its findings [31].

In another study, the effectiveness and tolerability of Dronabinol, ondansetron, and their combination were compared for treating delayed CINV over a 5-day period in a double-blind, placebo-controlled trial. Both Dronabinol and ondansetron showed similar efficacy in treating CINV, and the combination therapy did not exhibit superior efficacy compared to the monotherapy. The trial allowed flexible dosing of Dronabinol after initial fixed doses, with a median dosage of 20 mg/day. However, the short duration of the trial constrains the conclusions regarding long-term effectiveness [32].

A pilot phase II clinical trial investigated the tolerability, preliminary efficacy, and pharmacokinetics of a cannabis-based medicine, containing both delta-9-tetrahydrocannabinol and cannabidiol, alongside standard therapies for CINV control. This randomized, double-blind, placebo-controlled trial included patients who experienced CINV despite standard anti-emetic treatment. The study found that cannabis-based medicine, when added to standard antiemetic therapy, was better tolerated and offered more effective protection against delayed CINV compared to a placebo. Though more AEs were reported in the cannabis group, none were serious. The study's limited sample size, however, is a significant limitation, suggesting the need for validation through a phase III clinical trial [33].

2. Pain Management:

The role of THC in managing cancer-related pain has been a focus of various studies, indicating its potential effectiveness where traditional pain management strategies fail. Notably, Nabiximols (1:1 THC/CBD ratio) and THC-based treatments have been extensively researched in this context.

Johnson et al. initially conducted a double-blind RCT in 2010, followed by an open-label continuation study with 43 patients to evaluate Nabiximols and THC-only sprays [34, 35]. In total, 39 patients administered a self-titrated spray containing THC/CBD, whereas 4 patients exclusively used a THC spray. They adjusted the dosage based on symptom relief or maximum dose and underwent regular evaluations to ensure safety, tolerability, and clinical efficacy. According to this study, the long-term utilization of THC/CBD spray was well tolerated, and there was no evidence of a decrease in its effectiveness in relieving cancer-related pain over a prolonged period. Furthermore, patients who maintained the usage of the study medication refrained from seeking an escalation in dosage of this or any other pain-relieving medication over time, thereby indicating the potential utility of adjunctive cannabinoids in managing cancer-related pain [35].

In 2014, Lynch et al. conducted a double-blind crossover trial comparing Nabiximols with a placebo [36]. The study found no significant difference in neuropathic pain relief between the groups, although five individuals reported effective pain relief with Nabiximols. Furthermore, Fallon et al. conducted a study comparing Nabiximols and a placebo, with a much larger sample size [37]. This research paper presents the results of two meticulously conducted phase 3 trials, which were double-blind, randomized, and placebo-controlled, aiming to assess the effectiveness of Sativex in relieving cancer-related pain. Study 1 involved 399 randomly assigned patients, while study 2 included the randomization of 216 patients. With a substantial number of participants, the researchers concluded Sativex does not provide analgesic effects compared to the placebo. However, it did show greater efficacy, particularly among younger patients and those from the United States, suggesting a potential divergence in the effectiveness of cannabis [37].

Cote et al. analyzed the effects of Nabilone (a synthetic cannabinoid) versus placebo on overall QoL in a double-blind RCT [38]. The study was confronted with a significant rate of participant dropout, as 24 out of 56 patients withdrew, resulting in an impact on the trial's capacity to reach conclusive results. The results demonstrated that Nabilone did not improve the QoL, although it was well-tolerated and safe in patients undergoing treatment for head and neck cancers.

Zhang et al. published a prospective, case-matched cohort study [39]. A total of 74 patients diagnosed with head and neck cancer who were recreational marijuana users exhibited discernible differences in their QoL compared with 74 non-users. These differences encompassed reduced levels of anxiety, pain, and depression, alongside increased appetite and overall feelings of well-being, as measured by the Edmonton Symptom Assessment System and EuroQol-5D questionnaires. It is important to note that this study had limitations due to the design of the trial, as a QoL measures were only collected at baseline and did not monitor symptoms throughout the treatment period [39].

In 2012, one of the first randomized clinical trial investigated the combined effects of opioids and cannabinoids in adult cancer patients with moderate to severe pain, despite opioid treatment. Patients received Nabiximols in varying doses or a placebo over five weeks. The study, involving over 250 patients, found the most significant pain reduction in the low-dose group, with a decrease of 1.6 points on the Numeric Rating Scale (NRS) [40]. Later, a larger phase III trial compared Nabiximols to placebo in opioid-treated cancer patients but found no significant difference in pain reduction, although a numerical decrease of 0.8–0.9 points on the NRS was noted [41]. Other phase three trials echoed these findings, with Nabiximols showing no distinct advantage as a co-analgesic in high opioid-requiring cancer patients [42]. Contrastingly, a 2019 systematic review concluded that cannabinoids did not enhance pain relief or reduce opioid usage in cancer patients [43]. The recent MedCan1-CBD phase IIb study further supported this, finding that cannabidiol oil does not decrease opioid use in cancer patients [44]. Despite these findings and numerical improvements in pain scores, medical cannabis and cannabinoids have received only a "weak recommendation" for chronic cancer and non-cancer pain management in consensus statements [45].

Research involving interviews and questionnaires to explore the effects of cannabis on a range of symptoms has consistently indicated its substantial benefits. Bar-Sela's research revealed the broad applicability of cannabis, showing improvement in symptoms like nausea, vomiting, mood, fatigue, weight loss, and others related to cancer and its treatment [46]. The study conducted by Bar-Lev Schleider examined the safety and effectiveness of medical cannabis in a vast and diverse group of cancer patients [47]. The data was routinely collected as a component of the treatment program for 2970 cancer patients who received medical cannabis. Following a six-month period of follow-up, it was found that 95.9% of respondents experienced an improvement in their condition [47]. Despite the likelihood of selection bias in survey completion, these studies underscore the potential palliative benefits of medical cannabis for cancer patients, emphasizing the need for more prospective randomized trials to better understand the patient population and symptoms most positively impacted by cannabis use.

3. *Cancer*-related Cachexia and Anorexia Syndrome:

Cancer-related cachexia and anorexia syndrome (CACS) is a severe condition characterized by significant fat and muscle loss in advanced cancer patients, affecting 50–80% of this population and contributing to over 20% of cancer-related deaths [48, 49]. Beyond weight loss, CACS involves anemia, electrolyte imbalances, and anorexia, not solely caused by reduced food intake [50]. The syndrome's complexity extends from physiological changes to behavioral and psychological distress, significantly impacting patients' quality of life [51].

Management of CACS is challenging due to low patient adherence to exercise and nutrition interventions, and the limited efficacy and side effects of pharmacological treatments [51, 52]. Cannabis, historically used as food, has been suggested as a potential treatment for CACS, particularly for its appetite-stimulating properties through cannabinoid receptors in the CNS [53–55]. Clinical trials with cannabinoids like THC have shown improvements in appetite and weight stabilization in some cancer patients [56–60]. Nonetheless, the existing evidence regarding the effectiveness of cannabinoids in CACS is still considered being of low to very low, underscoring the imperative for further investigation [61].

4. Antitumor Effects:

The potential antitumor effects of THC are predominantly supported by preclinical studies, with substantial evidence emerging from laboratory and animal research. However, clinical evidence in humans remains sparse. A comprehensive review of previous clinical studies and abstracts from the International Cannabis Research Society’s annual meetings over the past three years did not reveal additional studies substantiating the antitumor effects of cannabis [62].

The evidence of antitumor impact in patients mainly revolves around two studies focusing on glioblastoma multiforme (GBM). Guzman et al. conducted a notable study where nine patients with recurrent and progressive GBM, post-surgery and radiotherapy, were treated with intratumor THC injections [63]. The median survival in this group was 24 weeks, with two patients living nearly a year. Remarkably, three out of nine patients showed clinical and/or radiographic improvement, and two exhibited decreased tumor-cell proliferation on repeat biopsies. This study suggested that direct THC injection into tumors might influence tumor growth and clinical outcomes. However, it is essential to note that this form of application differs significantly from typical cannabis consumption, thus limiting the generalization of these results to more common usage.

Twelves et al. conducted a randomized, placebo-controlled trial examining the use of Nabiximols in combination with temozolomide in patients with recurrent GBM [64]. In this trial, patients receiving the combination therapy exhibited improved 6-month progression-free survival, longer median survival, and higher 1-year survival rates compared to those receiving temozolomide alone. However, the study's small sample size and the preliminary nature of the results necessitate caution in interpretation.

Although there are numerous anecdotal claims and online assertions regarding the notable antitumor effects of cannabis, the direct clinical evidence remains scarce. Two case reports have been documented showing a potential antitumor impact of cannabis – one involving relapsed/refractory acute lymphoblastic leukemia and another concerning pilocytic astrocytoma [65, 66].

5. Safety and Adverse Effects:

EBM rigorously examines the safety profile of cannabinoids, indicating its general tolerability but also highlighting several side effects. Common AEs associated with THC include dizziness, dry mouth, psychoactive effects, and, in some cases, nausea/vomiting, and somnolence. These effects tend to be dose-dependent, with higher doses of cannabis often associated with a greater frequency and intensity of AEs [40, 67, 68].

Cognitive side effects have also been noted in cannabis users. Johnson et al. reported a deterioration in memory and concentration in individuals receiving Nabiximols, in contrast to improvements in the placebo group [34]. Bar Sela et al.’s observational study echoed these findings, noting a significant worsening of memory in patients with prolonged cannabis use [46].

According to the National Academies of Sciences, Engineering, and Medicine, the safety profile of cannabis in the general population is well-documented. Extensive evidence has been reported linking cannabis use to various health risks. These include an increased likelihood of motor vehicle crashes, lower birth weight in offspring, the potential development of schizophrenia and other psychoses, as well as the worsening of respiratory symptoms. Moreover, there is moderate evidence suggesting a risk of overdose injuries and impaired cognition. Additionally, limited evidence indicates an increased risk of certain health issues, such as non-seminoma-type testicular germ cell tumors and acute myocardial infarction [69].

Precautions and contraindications have been set for the usage of cannabis. These include age restrictions (under 25), pregnancy, schizophrenia, compromised cardiac conditions, and a previous history of substance abuse. Furthermore, it is advised to avoid using cannabis in combination with potent CYP3A4 inhibitors, sedatives, and hypnotics [16].

## Cannabinoids in the era of multimodal treatment in oncology:

In the complex interplay between tumorigenesis and immune response, inflammation has a paradoxical function, serving as both a facilitator of tumor development and a provider of potent tools for the immune system to counteract tumor growth. The presence of immune cells alongside cancer cells within a shared microenvironment has been a known phenomenon since the nineteenth century [75]. However, the transformative concept of utilizing immune cells as a direct means of combating cancer has gained prominence only recently. Jennifer Couzin-Frankel, in a seminal article in Science magazine, underscored this paradigm shift by emphasizing that immunotherapy introduces a fundamentally different approach to cancer treatment. It diverges from traditional methods by focusing on the immune system instead of directly targeting the tumor [76].

In the TME, CD8 + T cells collaborate with other immune constituents to target and eradicate tumors. However, a notable challenge arises during tumor progression when these immune cells, particularly cytotoxic CD8 + T cells, start losing their ability to destroy cancer cells effectively. This phenomenon has ushered in a new era of cancer treatment through the advent of targeted therapies, namely immunotherapy. Immunotherapy reinvigorates immune cells, removing the 'brakes' that hinder their tumor-fighting capabilities. This approach is categorized into two main types: active therapies, such as cancer vaccines, and passive therapies, including monoclonal antibodies and adoptive cell transfer [22].

The functionality of immune cells, particularly the activation and exhaustion status of cytotoxic CD8 + T cells, is regulated by several immune checkpoint proteins. These include PD-1, CTLA-4 (CD152), TIGIT, TIM-3, LAG-3, and BTLA, among others [77]. The focus of contemporary Immune Checkpoint Inhibitor (ICI) therapies has been on the PD-1/PD-L1 and CTLA-4 receptors [78, 79]. The approval of a CTLA-4 blocking antibody, ipilimumab, for treating melanoma in 2011 marked a significant advancement in this field. This development was followed by the introduction of monoclonal antibodies targeting PD-1 (such as pembrolizumab and nivolumab) and PD-L1 (including atezolizumab, durvalumab, and avelumab). To predict the efficacy of ICIs, biomarkers like PD-L1 levels in tumor tissues, tumor mutation burden (TMB), and microsatellite instability (MSI) have been utilized [80].

In addition to the biomarkers that predict responsiveness to ICI therapy, various other factors can induce resistance to immunotherapy. These factors need careful consideration both before and during therapy [80]. They encompass a wide range of elements, including genomic variations within the tumor, the heterogeneity of the tumor itself, the characteristics of immune cells and the TME, interactions between host cells and cancer cells, and a multitude of other factors such as age, biological sex, diet, hormones, existing comorbidities, medications, and the gut microbiome [81].

The field of immuno-oncology is constantly striving to identify ways to circumvent the development of resistance to immunotherapy and to discover additional targets to improve patient survival. In this quest, the ECS, known for its immunomodulatory properties, emerges as a potential source of such targets. However, the exploration of the ECS's role in the immune-TME and the impact of cannabis or cannabinoids in immunotherapy is still in its nascent stages. Studies by Xiong and colleagues have revealed that cannabinoids, both exogenous (THC) and endogenous (AEA), detrimentally impact antitumor immunity by impairing the function of tumor-specific T cells through CB2 receptors. Their research also indicates that THC can reduce the effectiveness of anti-PD-1 therapy [82].

The effects of cannabis use during immunotherapy have shown varying results. For instance, one study observed that cannabis use during nivolumab therapy led to a reduced response rate without affecting progression-free survival (PFS) or overall survival (OS) [83]. Bar-Sela et al. conducted a prospective observational clinical study involving 102 advanced cancer patients, comparing cannabis users with non-users undergoing ICI treatment. The study found that cannabis users experienced a significant decrease in time to treatment progression (TTP) and OS compared to non-users, although cannabis reduced irAEs. The authors suggested cautious use of cannabis before and during ICI immunotherapy in advanced malignancies [60]. In contrast, a study by Waissengrin et al. reported that the concurrent use of medical cannabis with pembrolizumab in advanced NSCLC patients did not show detrimental effects. While TTP did not vary significantly between cannabis users and non-users, the median OS was numerically higher for non-users, though this did not reach statistical significance (p = 0.08) [84].

The reason why there is a diminished response to ICI when using cannabis could be due to the immunosuppressive effects caused by cannabinoids. These effects may potentially involve the activity of CB2 in immune cells or other cells in the TME.

The exploration of the synergistic effects of cannabinoids in conjunction with other cancer treatments opens a promising avenue for enhancing multi-modal oncology therapies. It is important to understand how cannabinoids can be seamlessly integrated into such therapies, potentially leading to more effective and personalized strategies for cancer treatment.

While some new anticancer drugs are being used as monotherapies for specific indications, it is increasingly likely that cannabinoid compounds will find their place as combinatory or adjunctive options alongside existing cytostatics. In this context, THC and CBD, currently under investigation in some studies as combination therapies, have shown preclinical efficacy in enhancing the effects of a range of cytostatics. These include vinca alkaloids, cytarabine, doxorubicin, mitoxantrone, carmustine, temozolomide, bortezomib, carfilzomib, and cisplatin [16, 85].

The mechanisms underlying these synergistic effects, however, are not fully understood. One study indicated that the enhancement of vinblastine's effect in resistant leukemia cells by cannabinoids involved THC- and CBD-induced downregulation of P-glycoprotein. Similarly, the synergistic effect over mitoxantrone in embryonic fibroblasts occurred through inhibition of ATP-binding cassette transporters (ABCG2) [86, 87]. Another study focusing on THC's impact on leukemia cells revealed reduced p42/44 MAPK activity as a mechanism for THC-enhanced cytostatic effects [88]. Additionally, numerous mechanistic studies have demonstrated a CBD-mediated increase in tumor cell susceptibility to proteasome inhibitors like bortezomib, doxorubicin, temozolomide, and carmustine [89–92]. For instance, CBD has been shown to enhance the uptake and toxicity of doxorubicin, temozolomide, and carmustine in glioma cells through increased TRPV2 activity and associated calcium influx, a finding also confirmed for doxorubicin in triple-negative breast cancer cells [91, 92]. The synergistic effect with bortezomib was further elucidated, showing that the combination of CBD and THC inhibits the expression of the immunoproteasome subunit β5i in multiple myeloma cells [90]. Additionally, the combination of CBD and THC induces autophagy-dependent necrosis in multiple myeloma cells and inhibits cellular migration by downregulating CXCR4 and CD147 expression [90].

Furthermore, several studies indicate that cannabinoid treatment increases glioma cells' sensitivity to ionizing radiation, as seen with combinations of THC and CBD and CBD with heat shock inhibitors [93, 94]. Increased radiosensitivity in CBD-treated glioma cells was confirmed in another study, with subsequent research identifying the inhibition of ATM kinase activity, crucial in DNA double-strand break repair, as the underlying mechanism [95, 96].

## Emerging trends in cannabinoid research in oncology

In the dynamic field of oncology, the exploration of cannabinoids as therapeutic agents is gaining momentum, reflecting a blend of scientific curiosity and clinical necessity. Trials like the BELCANTO (NCT06097533) are at the forefront, assessing the cannabis extract Avextra 10/10 for palliative care patients, hoping to alleviate symptom burden and enhance well-being​​ [97]. Similarly, an observational study by the State University of New York (NCT06037681) is delving into the cannabis, opioids, and tobacco usage among diverse cancer patients, enriching our understanding of pain management in oncology​​ [98].

The CanAroma trial (NCT05935891), spearheaded by the University of Minnesota, is evaluating the use of topical cannabinoid creams for hormone receptor-positive breast cancer patients, a novel approach to managing Aromatase Inhibitor-Associated Musculoskeletal Syndrome (AIMSS) [99]​​. M.D. Anderson Cancer Center's observational study (NCT05836857) is another endeavour, gathering data on cannabis and CBD usage by cancer patients, a testament to the evolving landscape of pain management strategies in cancer care ​​[100].

City of Hope Medical Center's interventional study (NCT05672342) is pioneering the use of CBD and THC for mitigating symptoms of CTH-induced peripheral neuropathy in breast and colon cancer survivors, possibly ushering in a new era of symptom control in oncology [101]​​. The ARISTOCRAT trial (NCT05629702) is a phase II study evaluating Nabiximols in combination with Temozolomide for recurrent glioblastoma, aiming to improve OS [102]​​.

To understand the impact of cannabinoids on the TME, the University of Colorado Denver's study (NCT05520294) is correlating cannabinoid use with tumor infiltrating lymphocytes in malignant melanoma, potentially unlocking new avenues in cancer immunotherapy​​ [103]. Patient-reported outcomes are also a key focus, as seen in M.D. Anderson Cancer Center's study (NCT04875286), which is investigating preferences for cancer pain management with THC, CBD, and opioids, echoing the patient-centered approach in modern oncology​​ [104].

Tetra Bio-Pharma's SERENITY trial (NCT04001010) is exploring the realms of synthetic THC/CBD for patients with advanced cancer and cachexia, aiming to enhance physical functioning and modulate cachexia progression, a step towards improving quality of life in advanced cancer stages [105]​​. The phase III trial (NCT03984214) is assessing Dronabinol for CTH-induced and tumor-related symptoms in advanced pancreatic cancer, a pursuit to ease the burdens of one of the most challenging cancers [106]​​

The CAFCARS trial (NCT03948074) is an innovative study seeking the most effective cannabis extract for managing cancer-related symptoms like nausea, pain, anxiety, and sleep disturbance, potentially revolutionizing symptom management in oncology [107]​​. These trials collectively represent a paradigm shift in cancer care, where the amalgamation of traditional and novel therapies could redefine the therapeutic landscape.

## Summary

The topic of cannabinoids in oncology is multifaceted and intricate, requiring a combination of EBM and patient-centered care. Moreover, this field is heavily influenced by complex regulatory and legal frameworks.

While there is a growing interest in cannabinoids for cancer treatment, their approval is currently limited to managing nausea and vomiting. The transition from real-world data to clinical practice is hindered by the absence of established best practice standards for cannabinoids. Nonetheless, cannabinoids show promise in alleviating cancer-related pain, particularly as co-analgesics when used early in treatment, and seem to moderately enhance patient well-being by improving symptoms like mood, appetite, and anxiety. The exploration of cannabinoids' potential to inhibit tumor growth continues to be a topic of considerable scientific inquiry. The debate surrounding the anti-tumor potential of cannabinoids is complex. Due to the extensive influence of the ECS on cancer progression, cannabinoids exhibit a paradoxical nature in oncology, simultaneously presenting potential therapeutic benefits and challenges.

Current research on THC in oncology is often limited by small-scale or observational studies. These limitations restrict the reliability of the conclusions drawn from such studies. To achieve more definitive results, there is an urgent need for larger-scale studies and RCTs. These rigorous study designs would provide robust evidence necessary to guide clinical practice [108]. However, challenges exist in designing studies that can accurately reflect real-world use of canabinnoids while maintaining the integrity and rigor expected in scientific research. The variability in THC preparations and the lack of standardized dosing further complicate these research efforts.

In oncology, patient preferences and experiences are integral to treatment decisions. Some patients may opt for THC due to its perceived natural origin and potentially lower side effects compared to traditional medications. This trend underscores the importance of considering patient choice in determining the appropriateness of THC as a treatment option. Moreover, assessing the impact of THC on the QoL and overall well-being of oncology patients is vital. The potential benefits of THC on symptom management and QoL need to be weighed against any AEs.

While cannabinoids are generally well-tolerated, there are potential side effects and safety concerns, particularly with long-term use, that need thorough understanding. Issues in standardization and quality control of THC preparations pose additional challenges in ensuring consistent quality and dosing in both clinical and research settings. In the realm of THC's role in oncology, numerous questions remain unresolved concerning its efficacy, optimal dosing, and long-term impacts. The variation in the proportions of major active ingredients, such as THC and CBD, in cannabis leads to diverse therapeutic effects that are suitable for various conditions. To illustrate, it is advised to use products with a higher THC than CBD ratio to alleviate symptoms of anorexia, nausea, and vomiting. Balanced THC and CBD ratios are recommended for managing insomnia and pain, while a lower THC than CBD ratio is suggested for addressing anxiety and depression [109]​​.

There has been a growing interest among cancer patients in the potential therapeutic effects of cannabinoids. Despite this interest, many clinicians remain hesitant due to the lack of definitive, evidence-based data. A 2018 study involving 400 oncologists revealed that 70% perceived significant gaps in the data concerning cannabinoid use in cancer patients, even though 80% had engaged in discussions with their patients about this topic. This hesitation likely stems from the perception that current data on cannabinoids as an alternative therapy is inconsistent and challenging to assess due to the limited number of randomized controlled trials available [110]. This cautious attitude was also observed in a recent systematic review encompassing 21 studies from the United States, Canada, Europe, Australia, and Israel. The extent of clinical knowledge about cannabinoids' effects varied among clinicians, but those with experience prescribing medical cannabis were generally less concerned about adverse effects [111]. Additionally, the lack of standardization in cannabinoid products used outside of standard recommendations complicates large-scale database analyses, making it difficult to reproduce data and identify potential clinical benefits.

Furthermore, the legal status of THC varies significantly across countries, which affects its research, clinical application, and accessibility. In regions where THC is still illegal, substantial barriers hinder the ability to study and utilize its effects in clinical settings. As a result, clinicians must carefully navigate the complex legal and regulatory landscape when considering THC for treatment plans.

## Conclusion

Given the evolving nature of the evidence base surrounding cannabinoids use in oncology, a cautious approach is recommended for both clinicians and patients. Physicians need to be up to date with both the positive and negative aspects of cannabis use in cancer patients to guide treatment plans effectively. They should carefully consider the potential benefits and risks, while also being aware of the legal status of THC in their respective jurisdictions.

## Institutional Review Board Statement.

Not applicable.

## Data Availability

No datasets were generated or analysed during the current study.
